# Challenges and facilitators to evidence-based decision-making for maternal and child health in Mozambique: district, municipal and national case studies

**DOI:** 10.1186/s12913-020-05408-x

**Published:** 2020-06-30

**Authors:** Celso Inguane, Talata Sawadogo-Lewis, Eusébio Chaquisse, Timothy Roberton, Kátia Ngale, Quinhas Fernandes, Aneth Dinis, Orvalho Augusto, Alfredo Covele, Leecreesha Hicks, Artur Gremu, Kenneth Sherr

**Affiliations:** 1 Department of Global Health, University of Washington, 1107 45th Street. NE, Suite 350, Seattle, WA 98105 USA; 2 Institute for International Programs, Johns Hopkins University,, Baltimore, MD USA; 3 National Directorate of Public Health, Ministry of Health, Maputo City, Mozambique; 4 Institute for International Programs, Johns Hopkins University, Maputo City, Mozambique; 5 Department of Community Health, Faculty of Medicine, Universidade Eduardo Mondlane, Maputo City, Mozambique; 6 Health Alliance International, Maputo City, Mozambique; 7 Health Alliance International, Seattle, Washington USA

**Keywords:** Evidence-based, Decision-making, Maternal and child health, Mozambique

## Abstract

**Background:**

The need for evidence-based decision-making in the health sector is well understood in the global health community. Yet, gaps persist between the availability of evidence and the use of that evidence. Most research on evidence-based decision-making has been carried out in higher-income countries, and most studies look at policy-making rather than decision-making more broadly. We conducted this study to address these gaps and to identify challenges and facilitators to evidence-based decision-making in Maternal, Newborn and Child Health and Nutrition (MNCH&N) at the municipality, district, and national levels in Mozambique.

**Methods:**

We used a case study design to capture the experiences of decision-makers and analysts (n = 24) who participated in evidence-based decision-making processes related to health policies and interventions to improve MNCH&N in diverse decision-making contexts (district, municipality, and national levels) in 2014–2017, in Mozambique. We examined six case studies, at the national level, in Maputo City and in two districts of Sofala Province and two of Zambézia Province, using individual in-depth interviews with key informants and a document review, for three weeks, in July 2018.

**Results:**

Our analysis highlighted various challenges for evidence-based decision-making for MNCH&N, at national, district, and municipality levels in Mozambique, including limited demand for evidence, limited capacity to use evidence, and lack of trust in the available evidence. By contrast, access to evidence, and availability of evidence were viewed positively and seen as potential facilitators. Organizational capacity for the demand and use of evidence appears to be the greatest challenge; while individual capacity is also a barrier.

**Conclusion:**

Evidence-based decision-making requires that actors have access to evidence and are empowered to act on that evidence. This, in turn, requires alignment between those who collect data, those who analyze and interpret data, and those who make and implement decisions. Investments in individual, organizational, and systems capacity to use evidence are needed to foster practices of evidence-based decision-making for improved maternal and child health in Mozambique.

## Background

### Background and rationale

The need for evidence-based decision-making in the health sector is well understood in the global health community [1, 2]. Such understanding was recognized as early as the 1970s in the United Kingdom [3], and in the 1990s for low- and middle-income countries [4]. Growing global attention on the need to use evidence for decision-making led to the 2004 Mexico Summit declaration, which called on governments “to establish sustainable programmes to support evidence-based public health and health-care delivery systems, and evidence-based health-related policies” [2]. Nevertheless, a gap persists between the availability of evidence and the use of that evidence. This represents a significant missed opportunity in poor utilization of research, but more importantly, in lost potential towards improving population health outcomes.

Much research already exists on evidence uptake. A 2014 systematic review looking at facilitators and barriers to uptake of evidence in policymaking, describes an “explosion of research in the area” [5], and increased attention has led to more studies on the matter. The barriers cited in this review include: [1] “availability and access to research” and “improved dissemination”, [2] “clarity”, “relevance” or “reliability of research findings”, [3] “having no time or opportunity to use research”, [4] “policymakers and other users’” lack of “research skills”, and [5] personnel and other “costs”. Conversely, the cited facilitators were: [1] “availability and access to research”, [2] “collaboration between researchers and policymakers”, [3] “clarity”, “reliability” and “relevance of research findings”, [4] research evidence “relationship with policymakers”, and [5] research evidence “relationship with researchers”. These findings are consistent with those found elsewhere [1, 6–8], and most have also been found in Mozambique [9–12]. However, most research on evidence-based decision-making has been carried out in higher-income settings, and most studies look at policymaking rather than decision-making more broadly.

**Fig. 1:**
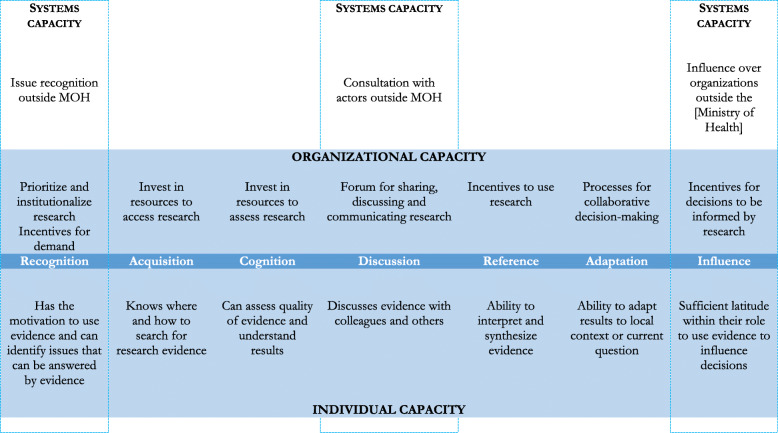
Conceptual framework for demand and use of evidence in the health sector - adapted [6]

There are numerous conceptual frameworks around transferring knowledge into action. Ward et al. found 28 different existing conceptual frameworks in a 2009 thematic literature analysis [1]**,** and more have been developed since then. Of particular interest for our study, Rodriguez et al.’s conceptual model (Fig. 1) was developed for Ministries of Health in low- and middle-income countries. It focuses on organizational, individual, and systems capacity, and on decision-making at all levels of the Ministry of Health, rather than strictly at policymaking level [6].

In Mozambique, at least two initiatives have been implemented to improve the use of evidence in decision-making in the health sector in recent years. At the provincial level, the Population Health Implementation Training Partnership in Africa (PHIT) implemented in Ghana, Mozambique, Tanzania, Rwanda and Zambia [13, 14] was implemented in all districts of Mozambique’s Sofala Province, from 2009 to 2015 [15]. The project aimed to improve the quality of routine data from the health information system (HIS) and promote the use of such data in decision-making processes about resource allocation, program monitoring, and improve service provision at the health facility and district levels [15]. At the national level, the National Evaluation Platform (NEP) project implemented from 2014 to 2018 in Mali, Malawi, Mozambique and Tanzania aimed to equip national government staff with the tools and skills to critically assess the quality of Maternal, Newborn and Child Health and Nutrition (MNCH&N) evidence from both routine and population-based sources, and promote the use of such evidence to influence policymakers and program leaders’ strategic decisions about MNCH&N at the country level [16–18]. Both initiatives aimed to change organizational culture around the use of evidence in decision-making.

We undertook this study to identify persisting challenges and facilitators to evidence-based decision-making in MNCH&N at the local (municipality and district) and national levels in Mozambique. The study can contribute to broader discussions within global health, about health systems strengthening in resource-limited countries [19], such as Mozambique.

## Methods

### Study design

This study is based on a combination of case studies that capture the diversity of experiences of evidence-based decision-making processes about MNCH&N at different levels where decisions about health policy and interventions are made in Mozambique (national, district and municipal), and at different performance levels on select maternal and child health indicators over time. The indicators were maternal and under-five mortality ratios between 1997 and 2011, because those were used in the most recent analysis published by the NEP in Mozambique [18]. We selected six cases: the NEP; Maputo City, two districts of Sofala Province (Caia and Chemba) and two of Zambézia Province (Alto Molócuè and Gilé). The NEP is an example of efforts to improve evidence-based decision-making at the national level, while Sofala is a similar example at the provincial, district and facility levels. Maputo City has consistently had lower maternal and under-five mortality rates compared to other provinces, is a municipality and the capital city of the country. Zambézia has consistently had the highest maternal and under-five mortality rates [18]. Our analysis period ranged from 2014 to 2017, the period of the first phase of the NEP, which overlaps with PHIT project activities. Study investigators relied on Sofala and Zambézia Provincial Directorates of Health (DPS) to select one district they regarded as a good example of evidence-based decision-making and one that had challenges with that process.

### Sampling

We sampled key informants who had participated in the generation of evidence and decision-making for planning and implementation of interventions aimed to improve reproductive maternal and under-five survival in each of the case studies, between 2014 and 2017, and continued in their institutions or were still associated with NEP activities at the time of interview. Although NEP activities were formally concluded in 2016 when the National Health Observatory was created [20], in practice, activities continued well into 2017–2018. We focused on key informants responsible for the production or assessment of evidence (analysts) and for decision-making (decision-makers) in the health sector and local government (districts and municipality). Often decision-makers in the Mozambican health sector are also program implementers. Therefore, this categorization of key informants is mostly for analytical convenience.

### Data collection methods

Over three weeks, in July 2018, two study investigators conducted in-depth individual interviews with key informants and reviewed documents on evidence-based decision-making about policy design, planning and implementation of interventions to reduce maternal and under-five mortality in each of the case studies, in the study reference period (2014–2017). Key informants were contacted through official letters and telephone and were interviewed at a place and time of their convenience, after obtaining their written informed consent. Interviews were audio-recorded whenever participants consented for the procedure. Study investigators documented each interview through notes, regardless of whether the interview was being recorded or not. Each participant was asked to share existing documents that could help understand evidence-based decision-making in their case study.

Study investigators conducted interviews using interview guides

(Additional file 1: interview guides)

tailored to each key informant category (decision-maker or analyst) and to each governance level (district or municipality and national level). Key domains in interview guides included describing decision-making processes (typology, regularity and key participants in decision-making, whether decision-making is evidence-based or not, and how those were documented), barriers and facilitators of evidence-based decision-making, and discussing examples of good practices of evidence-based decision-making. The document review guide helped prepare short annotations of each document (about 150 words), that described the document typology, key issues addressed in the document, institutions involved in decision-making or in the analysis and generation of evidence.

### Data analysis

To keep track of emerging themes, before leaving each site, study investigators prepared case-specific memos, using interview notes and document review annotations. Thematic analysis was conducted in ATLAS.ti, version 8.4 (Scientific Software Development GmbH), using constructs from the conceptual framework for the Ministry of Health’s capacity to demand and use research evidence (Fig. 1) [6]. The framework defines “[Ministry of Health] capacity to demand and use research evidence to inform policy and management decisions operating on three levels, namely, individual, organizational and systems levels” [6]. Whereas the framework focuses on research evidence, we focus on evidence both from population-based surveys and routine data from the HIS, because those are the main types of evidence that are mostly used in the Mozambican National Health System. We also apply the framework to the Ministry of Health at the central level and to the health portfolio at the local level (district and municipality).

The framework breaks-up demand and use of evidence into seven sequential steps, namely, “recognition, acquisition, cognition, discussion, reference, adaptation, and influence”. Recognition refers to “individual motivation to use evidence and [ability] to identify questions that can be answered by [ …] evidence”. Acquisition describes individual knowledge about where and how to search for evidence. Cognition refers to the ability to “assess the quality of evidence and understand results”. Discussion relates to sharing and discussing “evidence with colleagues, researchers and others”. Reference describes the “ability to interpret and synthesise [ …] evidence”. Adaptation is the “ability to adapt results to local context or current questions”. Influence describes people having “sufficient latitude within their role to use evidence to influence decisions” [6]. Demand of evidence encompasses recognition and acquisition, while the use of evidence covers the remainder steps. Individual capacity is expressed through individual skills to identify, assess and interpret evidence, distributed across the seven steps of evidence demand and use. Organizational capacity is manifest in Ministry of Health (and the health portfolio at various levels) “structures, practices, and resources that support the demand and use of research evidence in its decisions”. Systems capacity is reflected in “processes through which the [Ministry of Health] addresses the broader policy environment, and influences society and organizations beyond the [Ministry of Health]”. Individual and organizational capacity are reflected in all seven steps, while systems capacity is reflected in recognition, discussion and influence [6].

This thematic analysis process, followed a case-oriented approach [21] that focused on describing the characteristics of each case study and an extended case-study approach that captured similarities and differences across the cases [22]. Preliminary findings from the case-oriented approach were used to prepare case-specific reports that were shared with key informants for feedback. Findings presented in this manuscript reflect the extended case study approach.

### Ethical considerations

The study was approved by Mozambique’s National Bioethics Committee for Health (CNBS) and the Ministry of Health, after endorsement from the Directorates of Health of Maputo City, Maputo Municipality, and Sofala and Zambézia Provinces. Interviews were conducted after obtaining written informed consent from key informants. They consented separately for documenting the interviews using field notes and audio-recording. Audio-recordings and fieldnotes were protected using alphanumeric individual codes that replaced the identification of each key informant. Before preparing this manuscript, study investigators obtained key informants’ feedback on preliminary study findings, and that feedback was incorporated into the current manuscript.

## Results

### Sample characteristics

We interviewed 24 key informants, including 10 from the NEP (national level), two from Maputo Municipal Directorate of Health, and three from each of the two districts of Sofala and Zambézia Provinces (Table 1).

**Table 1:** Interviewees by category, at the national, district, and municipal levels, Mozambique, 2014–2017

Interviewee category	NEP	Maputo City	Caia	Chemba	Molócuè	Gilé	Total
Decision-maker	2	1	2	2	2	2	11
Analyst	8	1	1	1	1	1	13
Total	10	2	3	3	3	3	24

At the NEP we included two key informants from the steering committee (decision-makers) representing the home institution (INS) and the National Directorate of Public Health (DNSP). The remainder eight key informants from the Technical Working Group (TWG) were from the home institution, the National Directorate of Public Health and the National Directorate of Planning and Cooperation of the Ministry of Health, and from the National Institute of Statistics and the Ministry of Economics and Finance. We also interviewed a technical assistant from the NEP donor (Canada Department of Foreign Affairs Trade and Development) and another one from Universidade Eduardo Mondlane (UEM) a local public university. The two key informants from Maputo Municipality were the Council member responsible for the health portfolio (a decision-maker) and the statistics and planning supervisor (analyst).

At each district of Sofala and Zambézia we interviewed all key informants we had planned for, namely, a district government official, a decision-maker and a statistics and planning supervisor from the District Health, Women and Child Welfare Service. Because of time constraints, we could not interview two additional decision-makers from either the National Institute of Statistics, the Ministry of Education or of Economy and Finance, or from the Technical Secretariat for Food Security. In Maputo City, we could not interview a decision-maker at the municipal government, and at the provincial level we could not interview a government decision-maker, a health directorate decision-maker and a health analyst.

### Demand and use of evidence

**Fig. 2:**
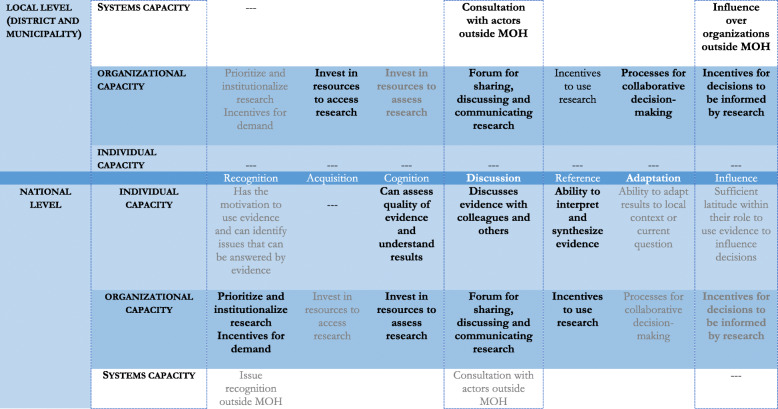
Demand and use of evidence for decision-making, at the national and local level, Mozambique, 2014–2017

At the district and municipal level and the national levels, the most salient domain was a high-quality organizational capacity to support the demand and use of evidence from routine HIS data and population-based surveys to inform decision-making. In Fig. 2, we compare constructs of individual, organizational, and systems capacity at the national level with the district and municipal level. The most prominent constructs are bolded, the less prominent ones are in grey, and those not mentioned are represented by dashes.

### District and municipal level

At the district and municipal levels, organizational capacity was more vocalized than systems capacity, while individual capacity was not visible in any step of the evidence-based decision-making process. Organizational capacity manifested mostly in the form of resources to access evidence from routine HIS data and population-based surveys (acquisition). In Maputo City, these investments in acquisition were also made on research studies. Regular health sector and local government meetings were cited as forums for sharing and discussing (discussing) and for collaborative decision-making (adaptation) in which actors outside the health sector, particularly representatives of local communities and international NGOs participated. This was more prominent in the two districts of Sofala, as a key informant noted:

- For instance, the involvement of the [local] government in decision-making. Here we plan for the [maternal and child lifesaving] interventions. Another example is the building of a new health facility, where different actors were involved, and we had to be based on evidence to justify building that health facility. [The district] had a low coverage of family planning consultation, and we had to include more actors and we trained several technicians and teachers to increase the coverage of family planning (Sofala, key informant, July 13, 2018).

The use of routine HIS data (across all cases) and of research (Maputo City) to make decisions, during local government and health-sector meetings, about building health facilities, improving training or allocating human resources to specific areas, were incentives for decisions to be informed by evidence (influence). Maputo City stood out for investment in prioritizing and institutionalizing research and providing incentives for its demand (recognition). This site was an exception in taking advantage of investments in resources for assessing the quality of evidence and understanding it (cognition), which a key informant explained that results from the city being better endowed with resources than the rest of the country. Specifically, Maputo City has higher qualified personnel, health facilities are located within a shorter radius from each other and have computers and better telecommunications network infrastructures, which ensures connection to the electronic-based HIS.

Across all local level cases, organizational capacity did not translate into individual abilities for demand and use of evidence. However, systems interactions were mentioned, especially local government and health authorities inviting representatives of other government sectors, community leaders, and international NGOs to bi-annual and annual meetings, for consultations (discussion), during which they obtained support to implement decisions (influence).

### National level

At the national level, organizational capacity was the most cited capacity, similarly to the local level, followed by individual capability and some systems capacity. NEP’s steering committee, by following a process of defining questions that the TWG had to answer by evaluating evidence from population-based surveys and routine HIS data, successfully institutionalized demand for research evidence (recognition). The NEP also invested in building the capacity of TWG members to assess evidence (cognition), to discuss their findings through regular meetings (usually monthly) and to share and discuss them with steering committee members (discussion), and to interpret and synthesize evidence. All this was supported by high-quality training provided by national and international experts, both in Mozambique and abroad, and by steering committee members’ insistence on the need for research excellence rather than on using evidence to inform decision-making.

This investment translated into TWG members’ individual capacity to assess the quality of evidence (especially routine data) and understand the results of routine and population-based survey data (cognition). TWG members also developed abilities to discuss evidence among themselves, during their meetings (usually monthly) and to present them to the steering committee, usually once to twice a year (discussion). TWG members’ ability to interpret and synthesize evidence (reference), is partially obvious in a summary the National Health Observatory published in 2017, which “identified patterns of seasonality in routine data to inform health policy and programs” (Summary 2, volume 1, October 2017). As a study participant noted, this individual capacity was also beneficial to their organization, the Ministry of Health:

- That capacity [to conduct modeling and develop analysis to inform decision-making] continues there [at the Ministry of Health]. The National Health Institute is highly motivated to lead the process. To lead research (NEP, key informant, July 9, 2018).

Albeit to a lesser extent, systems capacity for recognition and discussion, was expressed in the NEP. The multisectoral composition of the NEP’s steering committee and TWG helped raise awareness about questions the project addressed among government actors outside the MOH (recognition). Meetings of both bodies of the NEP were excellent forums for multisectoral consultation and learning (discussion) that particularly motivated TWG members, as one of them described:

- What motivated me the most in NEP was its multisectoral composition (Ministry of Economy and Finance, National Institute of Health, Technical Secretariat for Food Security, its multidisciplinary composition (physicians, public health specialists, biologists, statisticians) and people in different professional positions […]. It was a united class, which had no silos (NEP, key informant, July 3, 2018).

### Challenges and facilitators

More facilitators than challenges for evidence-based decision-making were mentioned at the district and municipal level. Conversely, more challenges were mentioned at the national level.

### District and municipal level

At the district and municipal level, both facilitators and challenges were for evidence use, rather than for demand (Table 2). Facilitators were at the organizational and systems levels, while challenges were at the systems level alone.

**Table 2:** Challenges and facilitators to use evidence for decision-making, at district and municipality levels, Mozambique, 2014–2017

	Facilitators	Challenges
***Organizational***	Access to evidence	
	Collaborative decision-making forum and process	
	Linkages between planning-implementation	
***Systemic***	Logistical and financial assistance from non-health sector actors	Top-down decision-making culture Lack of national government funding

The most prominent organizational level facilitators were access to electronic-based routine HIS data; collaborative decision-making at health-sector regular meetings in which non-health actors from the local government, community and NGO representatives were often invited; and linkages between planning of activities based on the available evidence and the ability to implement decisions made in those meetings. Systems level facilitators to use and demand of evidence were international NGOs’ funding and logistical support, and local government assistance, especially by allocating human resources and building health facilities.

- Access to evidence of quality, collaborative participation, development of planning and implementation capabilities […]. It is through these means that correct decisions are made, i.e., evidence is needed, collaboration of all involved in the decision-making process, those involved in the process of develop capabilities for better decision-making, which are then planned and implemented (Sofala, key informant, July 13, 2018).

The main challenges to the use of evidence were systemic. To a small extent, the lack of national government funding prevents the implementation of decisions at the local level. To a larger extent, however, a top-down approach to decision-making that relies on the central government and health sector planning documents, hinders decision-making sensitive to local specificities. This was noted by key informants in both Maputo City and a district in Zambézia Province.

- Look at how Maputo City looks likes from 7 am though 3:30 pm. It has nearly two million people. However, the planning was designed for the nearly 1.2 million inhabitants that the national census found. Another problem is how to reach Maputo City’s population. The greatest challenge here lies in looking at Maputo City as an exception to the rule. We need services appropriate to this reality. We have presented our concerns to the Ministry of Health, because this is the ministry that has to take adequate measures (Maputo City, key informant, July 12, 2018).
- We make central level decisions. In reality, we are more implementers than decision-makers. All decisions that are or were made are based on the five-year [central] government plan, which is the guiding document from which we create the Economic and Social Plan and the Health Sector Strategic Plan (Zambézia, key informant, July 24, 2018).

### National level

At the national level, key challenges were for use of evidence, and were found at the individual, organizational, and systemic levels, while the main facilitators were for demand of evidence and were at the organizational level alone (Table 3).

**Table 3:** Challenges and facilitators to demand and use evidence, at the national level, Mozambique, 2014–2017

	Facilitators	Challenges
**Demand**		
***Organizational***	Investment in human resources capacity building	
	Leadership engagement	
	Flexible communication strategies	
	Effective team building	
**Use**		
***Individual***		Human resource qualifications
***Organizational***		Mistrust of routine HIS data Weak dissemination and advocacy
		International partners impose global priorities over national priorities
***Systemic***		Limited central government funding availability
		Limited participation of non-MOH actors

The main individual challenge was limited qualified human resources in the public health sector and a training model focused on short-term sessions (week-long at most) that participants perceived to focus on building TWG members’ capacity to address the questions raised at the NEP, instead of capacities that could be applied to other questions and health issues they face in their everyday work. At the organizational level, participants perceived that dissemination of the results produced by the NEP was relatively later than needed and was not assertive enough. That the report published out of the work of the NEP did not include evidence from routine data made some participants perceive it as confirmation of the mistrust of quality of routine data that discourages its use in decision-making. Participants also perceived the influence of international NGOs and donors over the Ministry of Health as perpetuating decision-making practices that promote global priorities over national ones and prevent the institutionalization of evidence-based decision-making at the Ministry. A point made as follows:

- There is an informal NEP that influences decision-making at the Ministry [of health]. Such NEP is made up of [international] NGO technical staff who influence the Ministry’s programs and operational plans. That creates an important challenge to an evidence-based decision-making process, since it fragments priorities based on the influence of [international] NGOs and other partners of the Ministry of Health (NEP key informant, July 9, 2018).

At the systemic level, key informants regarded the limited central government funding as a key challenge to countering the influence of international organizations and priorities. They also noted that the lack of participation of non-health actors (representatives of Mozambican civil society organizations, private sector and international donors and NGO’s) in the NEP was a challenge to promoting decision-making based on evidence the NEP had produced.

Key facilitators were institutional investments in resources for capacity building of NEP’s TWG members in assessing the quality of evidence, analysing and synthesizing evidence, which was possible given the leadership of the steering committee, and creative communication and team building strategies that the project coordinators employed. This partially reflected in the following remarks:

- Finally, the good work environment created by the good relationships among the institutions involved and the search for regular collaboration, along with the strategic vision of the steering committee was very helpful (NEP key informant, July 4, 2018).

## Discussion

Our research into challenges and facilitators to evidence-based decision-making in MNCH&N at the municipality, district, and national levels in Mozambique suggests limited demand for evidence to inform decision-making. This may reflect local health officials not feeling empowered to make decisions, given a top-down approach in which decisions are made at the central level, while implementation occurs at the local level. The little mention of individual capacity for demand and use of evidence is consistent with this top-down approach because it reduces local level officers to implementers of plans and decisions that have been made elsewhere. It is also consistent with previous studies, which show that disengagement from the research process can lead to health officers feeling that policies designed centrally are not relevant to local realities [5, 23, 24]. Therefore, close collaboration between planners, decision-makers, and implementers at various levels, is key for overcoming this challenge; since it increases the likelihood that evidence is used at various levels and helps address the feeling of “collecting data for data’s sake” [5].

At the national level, it seems that all steps within the domains of demand and use of evidence identified in Rodriguez and colleagues’ framework were occurring at the organizational level. Conversely, none of those steps were present around the individual competences at the district and municipality levels, which echoes local concerns about the lack of individual research capacity. A key facilitator for developing data-use capacity that fosters multi-institutional collaboration is the positive feedback loop that occurs when individuals can see an immediate benefit to themselves or their institutions. This was the case of capacity building in the NEP, where the audience for findings was the steering committee [25]. Despite lacking individual competencies for demand and use of evidence, the district and municipality levels showed a tendency to connect organizational and systems capacity, by involving non-health, civil society and community actors in sharing and discussing evidence and in decision-making, which was not evident at the national level. This was done by using different data, including those from the routine HIS.

This resonates with lack of trust in evidence having also been identified as a barrier to evidence-based decision-making [24], at the national level but not at the district or municipality level. The hesitation to rely on HIS data, because of its perceived low quality, suggests that some data sources are not considered fit for use at the national level. While a healthy scepticism of poor-quality data is important for reliable analysis and interpretation, routine data has been shown to have quality [12, 26], which suggests that a systemic scepticism of data in general can be counterproductive. Future initiatives could seek to build capacity in data quality assessment and data ‘fluency’, such that actors recognize the value in all available data when well understood and appropriately interpreted.

Study participants reported that the influence of international actors weakens the capacity of the Ministry of Health to set its own priorities. Previous studies conducted in Mozambique also suggest that, if not carefully coordinated, external NGOs – who are often well-funded and equipped to implement their programs, but who operate with their own agendas – may foment fragmentation of the national primary health care system and undermine the country’s ability to set its own priorities [27, 28]. Conversely, the limited participation of non-Ministry of health actors weakens the potential influence of evidence outside the health sector. Advocacy and dissemination of results are important tools to address this and build an environment where evidence-based decision-making is both possible and encouraged.

### Limitations

The study missed perspectives from key informants who were not available for interview during the data collection period, especially some from the NEP’s steering committee and Maputo City’s provincial government. Because decision-making about health in Maputo City is under the responsibility of the municipality, this limitation has likely little influence over the study findings regarding Maputo City. Secondly, we did not have access to several documents, because they were not readily available or key informants did not feel comfortable sharing them without authorization from their supervisors. This challenge is ubiquitous in Mozambique [29] and can lead to the irony of not helping assess the extent to which the very goals that justify health interventions are met [30].

Notwithstanding these limitations, this study has ethical and methodological strengths. Specifically, the ethical approach we used, allowed us to fulfil the ethical obligation of reporting preliminary findings to study participants, and incorporate their feedback in this manuscript. The study design allowed us to describe facilitators and challenges that cover diverse contexts (district, municipal, and national levels) where evidence-based decision-making processes for maternal and child health occur in Mozambique.

## Conclusion

We undertook this study to identify persisting challenges and facilitators to evidence-based decision-making in MNCH&N at the district, municipal and national levels in Mozambique, in 2014–2017. Evidence-based decision-making requires that actors have access to evidence and are empowered to act on that evidence. This requires alignment between those who collect data, those who analyse and interpret data, and those who make and implement decisions. Such alignment is still absent in the cases we studied in Mozambique. Additionally, institutionalizing practices of evidence-use for decision-making requires engagement and empowerment at individual, organizational, and systems levels that still face challenges at various levels in Mozambique. Those challenges are related to the lack of balance between centralized decision-making and decentralized action, between national level goals and local level specificities, and between global priorities and national needs. Addressing those challenges is important to achieving maternal and child health outcomes grounded on reliable evidence-based decision-making in Mozambique.

## Data Availability

The data generated during this study are not publicly available for ethical reasons (to protect participant confidentiality, as stated in the consent form) but are available from corresponding author on reasonable request.

## Abbreviations

CNBS: Comité Nacional de Bioética para a Saúde National Bioethics Committee for Health
DNSP: Direcção Nacional de Saúde Pública (National Directorate of Public Health)
DPS: Direcção Provincial de Saúde (Provincial Directorates of Health)
HIS: Health Information System
INS: Instituto Nacional de Saúde (Nacional Insttute of Health)
MNCH&N: Maternal, Newborn and Child Health and Nutrition
NEP: National Evaluation Platform
NGO: Non-governamental Organization
ONS: Observatório Nacional de Saúde (National Health Observatory)
PHIT: Population Health Implementation Training Partnership in Africa
TWG: Technical Working Group
UEM: Universidade Eduardo Mondlane
